# ^1^H and ^13^C-NMR Data of the Simplest Plumeran Indole Alkaloids Isolated from *Aspidosperma S*pecies

**DOI:** 10.3390/molecules17033025

**Published:** 2012-03-09

**Authors:** Heloisa Alves Guimarães, Raimundo Braz-Filho, Ivo José Curcino Vieira

**Affiliations:** Laboratory of Chemical Science, State University of North Fluminense Darcy Ribeiro, 28013-602, Campos dos Goytacazes, RJ, Brazil; Email: hellofarma@gmail.com

**Keywords:** *Aspidosperma*, plumeran indole alkaloids, NMR spectral data

## Abstract

Indole alkaloids are the chemotaxonomic markers of the *Aspidosperma* genera. Those that have the simplest plumeran skeleton are classified as the precursors of biosynthetic routes and the intermediates for several synthetic reactions. This work aims to review the ^1^H and ^13^C-NMR data, up to 2011, describing the skeleton of 35 different plumeran indole alkaloids, from a group of 46 of them, and highlight the main spectral differences amongst them.

## 1. Introduction

The Apocynaceae family consists of 424 genera, divided into 17 tribes that are subdivided under five subfamilies: Rauwolfioideae, Apocynoideae, Asclepioideae, Periplocoideae and Secamonioideae [1]. About 400 species of Apocynaceae have been identified and grouped into 41 genera in Brazil, 78% of which are found only in the Amazonia region [2]. Several Apocynaceae are used for landscaping. These include the arboreal *Tabernaemontana* and *Plumeria*, the climbing *Alamanda* and *Mandevilla*, and the shrubby *Catharanthus* and *Nerium* [3]. The wood of some species is of particular commercial importance for local development. Species such as the popularly named “peroba” and “guatambu” are useful for making furniture and buildings [4].

The *Aspidosperma* genus is included in the Rauwolfioideae subfamily, Alstoniae tribe. The genus includes about 57 species, divided according to their chemotaxonomy into eight series—Rigida, Nitida, Quebranchines, Polyneura, Pyricolla, Nobile, Macrocarpa and Tomentosa, all of which are restricted to the American tropical and subtropical regions [5,6]. *Aspidosperma* is another genus of Apocynaceae of high commercial value due the good quality of its wood. 

Some species are also used in folk medicine; the infusion of its stem barks is used to treat a number of diseases [7]. For example, the extracts of the *Aspidosperma* stem bark, are characterized by the presence of indole alkaloids with high structural diversity. These alkaloids are responsible for the many pharmacological effects known for the plant [8]. The experimental assays [9,10,11,12,13] involving *A. ramiflorum* Müll. Arg., *A. pyrifolium* Mart., *A. megalocarpon* Müll. Arg., *A. macrocarpon* Mart. and *A. quebracho-blanco* Schltdl. species, attest to their popular use as an antileishmanial and an antimalarial. Alkaloids found in root bark of *A. ulei* Markgr. were used to evaluate and prove pro-erectile effects [14,15]. *A. subincanum* Mart., used in folk medicine to treat D*iabetes mellitus* and hypercholesterolemia [16], was proven to have a low acute toxicity in *in vivo* tests, resulting in its characterization as a non-toxic treatment [17]. *A. ramiflorum* Müll. Arg. exhibited antibacterial activity against the standard strains of Gram-positive (*Bacillus subtilis* and *Staphylococcus aureus*) and Gram-negative (*Escherichia coli* and *Pseudomonas aeruginosa*) [18] bacteria. The extracts of several different parts of *A. polyneuron* Müll. Arg. were tested against a wide range of fungi; only the ethanolic extract of the stem was able to inhibit the growth of *Cladosporium herbarum* [19].

The structural diversity of the indole alkaloids can be classified by examining their biosynthetic origins. The occurrence of complex alkaloids containing indole moieties is restricted to a few families; the best sources appear in the Apocynaceae, Loganiaceae and Rubiaceae families [20]. The indole alkaloids are *N*-methyl derivatives of tryptophan, which has a terpenic unit that originates from the mevalonic acid pathway. The metabolic origins of the remaining portion consist of a chain of ten carbon atoms, the sub-architecture of which is useful for dividing indole alkaloids into three classes: Iboga, Corynanthe and Aspidosperma. Usually, the C_9_ or the C_10_ chain units are shown to be of a terpenoid origin and are identified as secologanins (secoiridoids) [20,21].

**Figure 1:**
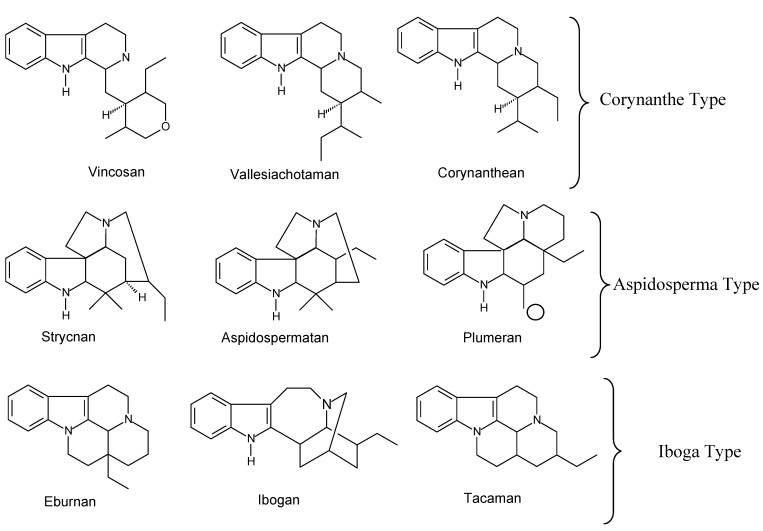
Subclasses of indole alkaloids.

Each of the three classes mentioned can be subdivided again, giving rise to nine other subclasses (Figure 1), according to Danieli and Palmisano [22]. Vincadifformine (**1**), as well as tabersonine (which presents a double bond between C-14 and C-15) are of Aspidosperma type. However, the loss of the carbon atom linked to C-16 (indicated by a circle and corresponding to the carboxylate function of secologanin) by hydrolysis/decarboxylation, originates most of the plumeran indole alkaloids [21].

The alkaloids isolated from *Aspidosperma* species have been exhaustively studied through phytochemical prospection, synthesis and semi-synthesis [23,24,25,26], as well the expression of secondary metabolites in cell cultures [27].

This work presents a review of the literature describing the ^1^H and ^13^C-NMR data of 35 alkaloids with a simplest plumeran skeleton. This alkaloid subclass was chosen for their structural diversity (the basic ring skeleton is representative of a large number of natural compounds) and the review of Pereira *et al*. (2007) [2] was adopted as a guideline. In this referred work, the criterion of classification is the same as that adopted by Manske [28]. Some structures (nomenclature and structure) were adjusted based on the literature. 

**Figure 2:**
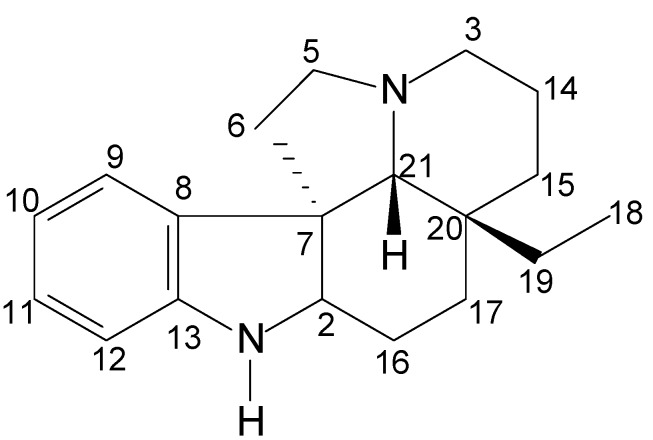
Numbering of the structure proposed by Le Men and Taylor [20].

The numbering of the structures in this work followed the method proposed by Le Men and Taylor (Figure 2) [20]. In many cases, it was not possible to obtain the NMR data, because the compounds’ structures were elucidated by other techniques (Infrared—IR, Ultraviolet—UV, Mass Spectrometry—MS).

**Figure 3:**
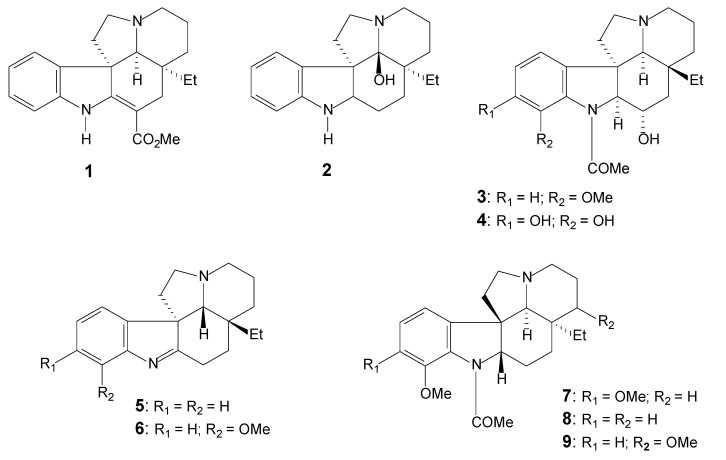
Plumeran indole alkaloids: methyl-*β*-anilineacrylate (**1**), fendlispermine (**2**), spegazzinine (**3** and **4**), aspidospermidine (**5** and **6**) and pyrifolidine (**7** to **9**) skeletons [28].

**Figure 4:**
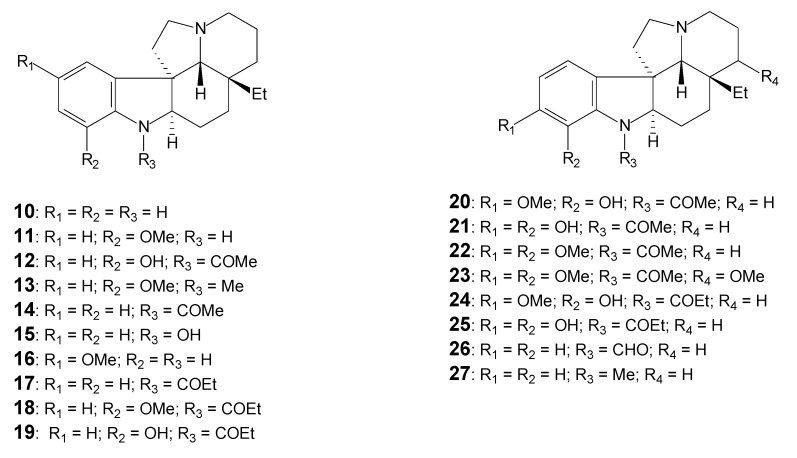
Plumeran indole alkaloids: Aspidospermine (**10** to **19**) and Aspidoscarpine (**20** to **27**) types [28].

**Figure 5:**
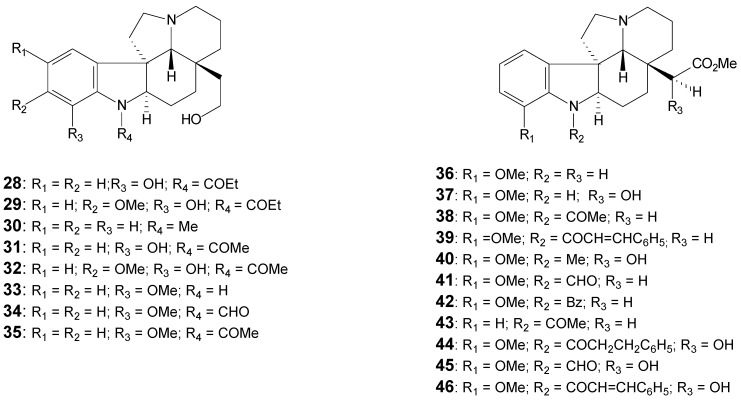
Plumeran indole alkaloids: Limaspermine (**28** to **35**) and Cylindrocarine (**36** to **46**) skeletons [28].

Table 1 lists the alkaloids, the respective plant origin and the spectral data available for each. The alkaloids listed are shown in Figure 3, Figure 4 and Figure 5.

**Table 1:** Plumeran indole alkaloids isolated from *Aspidosperma*.

Alkaloid	Species [2]	Data
(−)-Vincadifformine (**1**)	*A. macrocarpon, A. pyrifolium*	^1^H-NMR [29], ^13^C-NMR [30]
Fendlispermine (**2**)	*A. fendleri*	*
Spegazzinine (**3**)	*A. chakensis*	^1^H-NMR [31]
Spegazzinidine (**4**)	*A. chakensis*	^1^H-NMR [31]
1,2-Dehydroaspidospermidine (**5**)	*A. neblinae, A. quebracho-blanco*	^1^H-NMR, ^13^C-NMR [32]
1,2-Dehydro- *N*-deacetyl-aspidospermin (**6**)	*A. neblinae*	IR, UV, MS [33]
(+)-Pyrifolidine (**7**)	*A. cylindrocarpon, A. neblinae, A. pyrifolium,* *A. refractum, A. quebracho-blanco*	^1^H-NMR, ^13^C-NMR [32]
(+)-Aspidospermine (**8**)	*A. album, A. australe, A. exalatum, A. peroba,* *A. polyneuron, A. pyricollum, A. pyrifolium,* *A. quebracho-blanco, A. quirandy, A. sessiflorum, A. rhombeosignatum*	^1^H-NMR, ^13^C-NMR [34,35]
15-Methoxyaspidospermine (**9**)	*A. pyrifolium*	^1^H-NMR, ^13^C-NMR [32]
Aspidospermidine (**10**)	*A. neblinae, A. quebracho-blanco, A. pyrifolium, A. rhombeosignatum*	GC/MS [36,37]
Deacetylaspidospermine (**11**)	*A. neblinae, A. polyneuron, A. pyrifolium,* *A. quebracho-blanco*	^1^H-NMR [38]
(+)- *O*-Demethylaspidospermine (**12**)	*A. discolor, A. eburneum, A. excelsum,* *A. neblinae, A. pyricollum*	^1^H-NMR [39]
*N*-Methyl-deacetylaspidospermine (**13**)	*A. quebracho-blanco*	MS [40]
Demethoxyaspidospermine (**14**)	*A. discolor, A. macgravianum, A. neblinae, A. pyrifolium*	^1^H-NMR [39]
Aspidosine (**15**)	*A. quebracho-blanco*	IR, MS [41]
10-Methoxy-aspidospermidine (**16**)	*A. pyrifolium*	^1^H-NMR, ^13^C-NMR [42]
Demethoxypalosine (**17**)	*A. discolor, A. exalatum, A. limae,* *A. rhombeosignatum*	MS [39]
Palosine (**18**)	*A. polyneuron, A. pyrifolium*	^1^H-NMR, ^13^C-NMR [42]
*O*-Demethylpalosine (**19**)	*A. exalatum, A. limae, A. pyrifolium*	^1^H-NMR [43]
Aspidocarpine (**20**)	*A. album, A. formosanum, A. limae,* *A. marcgravianum,A.megalocarpon*	^1^H-NMR, ^13^C-NMR [44]
*O*-Demethylaspidocarpine (**21**)	*A. album, A. cuspa, A. melanocalyx*	^1^H-NMR [45]
Deacetylpyrifolidine (**22**)	*A. neblinae, A. quebracho-blanco*	GC/MS [46]
15-Methoxypyrifolidine (**23**)	*A. pyrifolium*	^1^H-NMR, ^13^C-NMR [32]
Aspidolimine (**24**)	*A. limae, A. obscurinervium*	^1^H-NMR [47]
*N*-Propionyl-16,17-dihydroxyaspidospermidine (**25**)	*A. melanocalyx*	GC/MS [48]
*N*-formyl-aspidospermidine (**26**)	*A. pyrifolium*	** [49]
*N*-Methylaspidospermidine (**27**)	*A. quebracho-blanco*	^1^H-NMR, ^13^C-NMR [50]
Limaspermine (**28**)	*A. limae*	^1^H-NMR [51]
11-Methoxylimaspermine (**29**)	*A. album*	^1^H-NMR [52]
Limaspermidine (**30**)	*A. rhombeosignatum*	MS [53]
Limapodine (**31**)	*A. album, A. limae, A. marcgravianum*	^1^H-NMR [52]
11-Methoxylimapodine (**32**)	*A. limae*	^1^H-NMR [52]
Cylindrocarpinol (**33**)	*A. cylindrocarpon*	IR, MS [54]
*N*-Formylcilindrocarpinol (**34**)	*A. cylindrocarpon*	^1^H-NMR [55]
*N*-Acetylcylindrocarpinol (**35**)	*A. cylindrocarpon*	^1^H-NMR [25]
Cylindrocarine (**36**)	*A. cylindrocarpon*	^1^H-NMR[23,25], ^13^C-NMR [23]
19-Hidroxycylindrocarine (**37**)	*A. cylindrocarpon*	^1^H-NMR [54]
Cylindrocarpidine (**38**)	*A. cylindrocarpon*	^1^H-NMR [25], ^13^C-NMR [32]
Cylindrocarpine (**39**)	*A. cylindrocarpon*	^1^H-NMR [56]
*N*-Methyl-cylindrocarine (**40**)	*A. cylindrocarpon*	^1^H-NMR [55]
*N*-Formyl-cylindrocarine (**41**)	*A. cylindrocarpon*	^1^H-NMR, ^13^C-NMR [23]
*N*-Benzoylcylindrocarine (**42**)	*A. cylindrocarpon*	^1^H-NMR [55]
12-Demethoxy- *N*-acetylcylindrocarine (**43**)	*A. cylindrocarpon*	^1^H-NMR, ^13^C-NMR [23]
*N*-Dihydrocinamoil-19-hydroxy-cylindrocarine (**44**)	*A. cylindrocarpon*	^1^H-NMR [55]
*N*-Formyl-19-hydroxycylindrocarine (**45**)	*A. cylindrocarpon*	^1^H-NMR [23]
*N*-Cinnamoyl-19-hidroxycylindrocarine (**46**)	*A. cylindrocarpon*	^1^H-NMR [23]

* Data not found; ** The alkaloid was identified by comparison, after the acetylation of aspidospermidine.

Table 2 shows the ^1^H-NMR data of some alkaloids. The remarkable feature is the typical aspidospermine signals of hydrogen linked to CH-2, whose chemical shift remains at *δ*_H_ 4.5. The multiplicity of this signal, a double-doublet, reveals the spin coupling of H-2 with the two hydrogen atoms at CH_2_-16 unsubstituted. This pattern is present in almost all alkaloids, except for those substituted in C-16vincadifformine (**1**, with double bond between C-2 and C-16) [30], spegazzinine (**3**) and spegazzinidine (**4**) [31], and also 1,2-dehydroaspidospermidine (**5**) and 1,2-dehydro-*N*-deacetyl-aspidospermin (**6**), although the NMR data for the latter is not available.

Table 2^1^H-NMR data (in CDCl_3_) for the plumeran indole alkaloids from *Aspidosperma* species. (Multiplicities, *J*, in parenthesis).

**Table:** 

Hydrogens		Compound/ *δ*_H_ (ppm), *J*_H_ (Hz)			
	1	3	4	5	7
**2**	-	4.05 ( *d*, 8.0)	4.05 ( *d*, 8.0)	-	4.60 (1H, *s*)
**3**				3.18 (1H, *m*)2.48 (1H, *dd*, 13.2; 3.3)	3.02 (1H, *d*, 11.0)1.96 (1H, *m*)
**5**				3.20 (1H, *m*)2.80 (*ddd*, 10.2; 3.3)	3.10 (1H, *dt*, 8.0)2.20 (1H, *m*)
**6**				2.18 (1H, *m*)1.56 (1H, *m*)	2.00 (1H, *m*)1.56 (1H, *m*)
**9**	6.74–7.5 (4H, *m*)	6.57 (2H, *m*)	≈7.0 (3H, *m*)	7.35 (1H, *d*, 8.0)	6.83 (1H, *d*, 8.6)
**10**	6.74–7.5 (4H, *m*)	6.57 (2H, *m*)	≈7.0 (3H, *m*)	7.25–7.30 (1H, *m*)	6.64 (1H, *d*)
**11**	6.74–7.5 (4H, *m*)		≈7.0 (3H, *m*)	7.25–7.30 (1H, *m*)	
**12**	6.74–7.5 (4H, *m*)			*7.*53 (1H,*d*, 8.0)	
**14**				1.86 (2H, *dq*, 12.9)1.52 (1H, *m*)	1.73 (1H, 4.0)1.51 (1H, *m*)
**15**				1.58 (1H, *m*)1.0 (1H, *dt*, 13.5; 2.7)	1.64 (1H, *m*)1.08 (1H, *m*)
**16**				2.60 (1H, *m*)3.1 (1H, *ddd*)	1.54 (1H, *m*)1.80 (1H, *m*)
**17**				1.28 (1H, *m*)1.47 (1H, *m*)	1.08 (1H, *m*)2.02 (1H, m*)*
**18**		0.75 (3H, *t*)	0.75 (3H, *t*)	0.50 (3H, *t*, 6.9)	0.63 (3H, *t*, 7.3)
**19**				0.65 (2H, *q*, 6.9)	0.81 (1H, *q*, 6.9)1.25 (1H, *m*)
**21**	8.96 (1H, *br s*)			2.42 (1H, *s*)	2.18 (1H, *s*)
**11-OCH_3_**					3.98 (3H, *s*)
**12-OCH_3_**					3.78 (3H, *s*)
**15-OCH_3_**					
**COCH_3_**		2.48 (3H, *s*)	2.48 (3H, *s*)		2.15 (3H, *s*)
**COOCH_3_**	3.76 (3H, *s*)				
**OH (C11)**		-	5.84 (1H, *m*)		
**OH (C12)**		11.1 (1H, *s*)	11.1 (1H, *s*)		
**OH (C16)**		7.3 (1H, *s*)	7.3 (1H, *s*)		
	1.6–3.6 (18H, *complex m*)

**Table:** 

Hydrogens	Compound/ *δ*_H_ (ppm), *J_H_* (Hz)						
	8	9	11	12	14	16	18
**2**	4,5 (1H, *m*)	4.70 (1H, *q*)	3.48 ( *q*, 6; 10)	4.07 ( *q*, 6; 10)	4.08 (1H, *q*, 6, 10)	3.6 ( *dd*, 6.2, 11)	4.5 ( *m*)
**3**	3.0 ( *brd*, 10.7) 1.9 (*m*)	3.28 (1H, *m*) 2.22 (1H)				3.0 ( *dt*, 13.1, 4.1) 1.95 (*dd*, 2.9, 10.7)	3.0 ( *brd*, 10.7) 2.05–1.9 (*m*)
**5**	3.11 ( *td*, 9, 3.2) 2.25 (*m*)	3.48 (1H, *m*) 2.40 (*m*, 13.8)				3.1 ( *td*, 7.6, 2.8) 2.3 (*m*)	3.1 ( *td*, 9, 3.2) 2.25 (*m*)
**6**	2.05 ( *m*) 1.55 (*m*)	2.20 (1H, *m*) 1.80 (1H, *m*)				2.4 ( *m*) 1.5 (*m*)	2.05–1.9 ( *m*) 1.6–1.35 (*m*)
**9**	6.83 ( *d*, 8.7)	6.82 (2H, 8.0)	6.56 (3H, *m*)	6.9 (3H, *m*)	7.17 (3H, *m*)	6.75 ( *d*, 1.7)	6.83 ( *d*, 8.7)
**10**	7.07 ( *t*, 8.7)	7.08 (1H, *d*, 8.0)	6.56 (3H, *m*)	6.9 (3H, *m*)	7.17 (3H, *m*)		7.07 ( *t*, 8.7)
**11**	6.8 ( *d*, 8.7)	6.82 (2H, 8.0)	6.56 (3H, *m*)	6.9 (3H, *m*)	7.17 (3H, *m*)	6.6 ( *dd*, 1.7, 7)	6.8 ( *d*, 8.7)
**12**					8.13 (1H, )	6.7 ( *d*, 7)	
**14**	1.73 ( *qt*, 13.4) 1.5 (*brd*, 13)	2.0 (1H) 1.60 (1H, *m*)				1.8 ( *qt*, 13.1, 4.0) 1.5 (*m*)	1.7 ( *qt*, 13, 4) 1.6–1.35 (*m*)
**15**	1.6 ( *m*) 1.05 (*m*)	3.22 ( *m*, 14.5, 7.0)				1.6 ( *m*) 1.2 (*dd*, 4.5, 13.4)	1.6–1.35 ( *m*) 1.3–1.05 (*m*)
**16**	1.95 ( *m*) 1.35 (*m*)	1.30 (1H, *m*) 2.04 (1H, *m*)				1.6 ( *m*) 1.4 (*dt*, 14.0, 3.4)	2.05–1.9 ( *m*) 1.6–1.35 (*m*)
**17**	2.0 ( *m*) 1.1 (*m*)	1.10 (1H, *dq*, 14.7) 2.18 (1H, *m*)				2.0 ( *dd*, 2.6, 12.3) 1.1 (*dt*, 12.6, 2.5)	2.05–1.9 ( *m*) 1.3–1.05 (*m*)
**18**	0.6 ( *t*, 7.1)	0.63 (3H, *t*, 6.9)	0.6 (3H, *t*, *6*)	0.72 (3H, *t*)	0.73 (3H, *t*)	0.7 ( *t*, 7.3)	0.6 ( *t*, 7)
**19**	1.2 ( *dq*, 14, 7.1) 0.8 (*dq*, 14, 7.1)	0.75 (1H, *q*, 6.9) 1.22 (1H, *m*)				1.5 ( *m*) 0.9 (*dq*, 14.3, 7.3)	1.3–1.05 ( *m*) 0.8 (*dq*, 14, 7)
**21**	2.23 (1H, *s*)	2.20 (1H, *s*)	2.16 (1H, *s*)	2.28 (1H, *s*)		2.2 (1H, *s*)	2.2 ( *s*)
**10-OCH_3_**						3.8 (3H, *s*)	
**12-OCH_3_**	3.87 (3H, *s*)	3.90 (3H, *s*)	3.78 (3H, *s*)				3.87 ( *s*)
**15-OCH_3_**		3.33 (3H, *s*)					
**COCH_3_**	2.2 (3H, *s*)	2.22 (3H, *s*)	-	2.32 (3H, *s*)	2.27 (3H, *s*)		
**COCH_2_CH_3_**							2.6 ( *dq*, 13, 6.5) 2.4 (*m*)
**COCH_2_CH_3_**							1.3 ( *t*, 6.5)
**OH**				10.83 ( *s*)			
**NH**			3.35 (1H, *s*)

**Table:** 

Hydrogens		Compound/ *δ*_H_ (ppm), *J_H_* (Hz)					
	19	20	21	23	24	27	28
**2**	4.07 ( *q*, 6, 10)	4.07 ( *dd*, 11, 6)	4.0 (1H, *q*, 5)	4.71 (1H, *q*)	4.12 (1H, *q*)	3.4 ( *dd*, 5.5, 9)	4.12 (1H, *q*)
**3**		1.98 ( *td*, 12, 4) 3.04 (*dm*, 12)		3.26 (1H, *m*) 2.20 (1H)		3.1–3.05 ( *m*) 1.8–2.0 (*m*)	
**5**		2.27 ( *m*), 3.12 (*m*)		3.48 (1H, *m*) 2.40 (*m*, 13.8)		3.1–3.05 ( *m*) 2.3 (*m*)	
**6**		1.57 ( *m*), 2.04 (*m*)		2.20 (1H, *m*) 1.80 (1H, *m*)		2.3 ( *m*) 1.4–1.55 (*m*)	
**9**	6.78 ( *m*, 8)	6.61 ( *d*, 8)	6.36 ( *s*)	6.65 (1H, *d*, 8.0)	6.65 (2H, *q*, 8)	7.0 ( *d*, 7.5)	6.92 (3H, *m*)
**10**	6.77 ( *m*, 7)	6.69 ( *d*, 8)	6.36 ( *s*)	6.82 (1H, *d*, 8.0)	6.65 (2H, *q*, 8)	6.6 ( *dt*, 1, 7.5)	6.92 (3H, *m*)
**11**	7.02 ( *m*, 1.5)					7.05 ( *dt*, 1, 7.5)	6.92 (3H, *m*)
**12**						6.35 ( *d*, 7.5)	
**14**		1.72 ( *tm*, 12) 1.53 (*dm*, 12)		2.10 (1H) 1.52 (1H, *m*)		1.75 ( *m*) 1.4–1.55 (*m*)	
**15**		1.11 ( *td*, 12, 4) 1.65 (*dt*, 12, 4)		3.22 ( *m*, 14, 7)		1.65 ( *m*) 1.1–1.2 (*m*)	
**16**		1.86 ( *m*) 1.52 (*m*)		1.25 (1H, *m*) 2.0 (1H, *m*)		1.75 ( *m*) 1.25 (*m*)	
**17**		2.00 ( *td*, 12, 14) 1.15 (*dm*, 12)		1.00 ( *dq*, 14.7) 2.15 (1H, *m*)		1.8-2.0 ( *m*) 1.1–1.2 (*m*)	
**18**	0.59 ( *t*, 14)	0.63 ( *t*, 7.5)	0.7 ( *t*, 4)	0.63 (3H, *t*)	0.62 (3H, *t*, 6)	0.6 ( *t*, 7.5)	3.52 ( *t*, 2H, 7)
**19**		0.93 ( *m*), 1.44 (*m*)		0.76 (1H, *q*, 7.2) 1.30 (1H, *m*)		0.85 ( *dq*, 14.5, 7.5) 1.4–1.55 (*m*)	
**21**		2.25 ( *s*)		2.28 (1H, *s*)		2.2 ( *s*)	
**11-OCH_3_**		3.88 ( *s*)		3.90 (6H, *s*)	3.88 (3H, *s*)		
**12-OCH_3_**				3.90 (6H, *s*)			
**15-OCH_3_**				3.22 (3H, *s*)			
**COCH_3_**		2.33 ( *s*)	2.25 ( *s*)	2.25 (3H, *s*)			
**COCH_2_CH_3_**	2.53 ( *q*, 14)				2.57 (2H, *q*, 7)		2.57 (2H, *q*, 7.5)
**COCH_2_CH_3_**	1.24 ( *t*, 14.2)				1.25 (3H, *t*, 7)		
**Ph-OH**	10.86 ( *s*)	10.98 ( *s*)	10.85 (1H, *s*)		10.98 (1H, *s*)		10.88 (1H, *s*)
***N*-CH_3_**						2.75 ( *s*)	
	0.3–3.2 (17 hydrogens)

**Table:** 

Hydrogens	Compound/ *δ*_H_ (ppm), *J*_H_ (Hz)							
	29	31	32	34	35 **	36 **	37	38 **
**2**	4.1 ( *q*)	4.1 (1H, *q*)	4.07 ( *q*)	*	5.5 (1H, *bq*, 5)	6.7–6.95 ( *m*)	3.60 (1H, *m*)	5.4 (1H, *m*)
**3**								
**5**					6.8–7.1 (2H, *m*)	6.7–6.95 ( *m*)		6.5–7.0 (2H, *m*)
**6**								
**9**	6.5–6.9 (2H, *q*, 8)	6.58–7.25 (3H, *m*)	6.5–6.9 (2H, *q*, 8)	6.6–7.1 (3H, *m*)	7.75 (3H, *m*)	6.8–6.61 (3H, *m*)	6.55–6.93 (3H, *m*)	7.57 (3H, *m*)
**10**	6.5–6.9 (2H, *q*, 8)	6.58–7.25 (3H, *m*)	6.5–6.9 (2H, *q*, 8)	6.6–7.1 (3H, *m*)	7.75 (3H, *m*)	6.8–6.61 (3H, *m*)	6.55–6.93 (3H, *m*)	7.57 (3H, *m*)
**11**		6.58–7.25 (3H, *m*)		6.6–7.1 (3H, *m*)	7.75 (3H, *m*)	6.8–6.61 (3H, *m*)	6.55–6.93 (3H, *m*)	7.57 (3H, *m*)
**12**								
**14**								
**15**								
**16**								
**17**								
**18**	3.55 (2H, *t*, 7)	3.53 (2H, *t*, 7)	3.53 (2H, *t*, 7)					
**19**					6.47 (2H, *bt*, 7)		4.31 (1H, *d*, 6)	
**21**	2.5 ( *s*)	*	*		2.85–3.3 (1H, *s*)	3.2–3.4	2.93 (1H, *s*)	2.8–3.2 (1H, *s*)
**11-OCH_3_**	3.87 ( *s*)		3.87 ( *s*)					
**12-OCH_3_**				2.30 (3H, *s*)	6.15 (3H, *s*)	6.18 (3H, *s*)	3.79 (3H, *s*)	6.13 (3H, *s*)
**18-OCH_3_**							3.88 (3H, *s*)	
**N-COCH_3_**		2.32 ( *s*)	2.32 ( *s*)		7.84 (3H, *s*)			7.80 (3H, *s*)
**N-COCH_2_CH_3_**	2.57 ( *q*, 7.5)							
**N-COCH_2_CH_3_**	1.27 ( *t*, 7.5)							
**N-COOCH_3_**						6.44 (3H, *s*)		6.44 (3H, *s*)
**N-CHO**				9.3 (1H, *s*)				
**Ph-OH**	10.95 (1H, *s*)	10.87 (1H, *s*)	10.95 (1H, *s*)					
**C-19-OH**							2.74 (1H, *d*, 6)	
					7.6–9.2 (15H, *m*)	7.4–8.9 (14H, *m*)		7.5–9.1 (14H, *m*)

**Table:** 

Hydrogens		Compound/ *δ*_H_ (ppm), *J_H_* (Hz)						
	39	40	41	42	43	44	45	46
**2**	4.50 (1H, *m*)	3.9–4.1 (1H, *q*)	4.54 (1H, *dd*, 10.5, 6.1)	4.24–4.44 (1H, *q*)	4.04 (1H, *dd*, 10.8, 8)	4.35–4.6 (1H, *q*)	*	4.50–4.70 (1H, *q*)
**3**								
**5**			3.07 (2H, *m*)		3.3-2.95 (2H, *m*)			
**6**								
**9**		6.56–6.75 (3H, *m*)	7.1–6.72 (3H, *m*)	6.6–7.6 (8H, *m*)	7.3–6.9 (3H, *m*)	6.7–7.2 (8H, *m*)	6.6–7.3 (3H, *m*)	6.8–7.6 (8H, *m*)
**10**		6.56–6.75 (3H, *m*)	7.1–6.72 (3H, *m*)	6.6–7.6 (8H, *m*)	7.3–6.9 (3H, *m*)	6.7–7.2 (8H, *m*)	6.6–7.3 (3H, *m*)	6.8–7.6 (8H, *m*)
**11**		6.56–6.75 (3H, *m*)	7.1–6.72 (3H, *m*)	6.6–7.6 (8H, *m*)	7.3–6.9 (3H, *m*)	6.7–7.2 (8H, *m*)	6.6–7.3 (3H, *m*)	6.8–7.6 (8H, *m*)
**12**					8.12 (1H, *m*)			
**14**								
**15**								
**16**								
**17**								
**18**								
**19**						3.98 (1H, *s*)	4.19 (1H, *s*)	4.06 (1H, *s*)
**21**	2.48 (1H, *s*)		2.46 (1H, *s*)	2.50 (1H, *s*)	2.53 (1H, *s*)		2.99 (1H, *s*)	3.00 (1H, *s*)
**12-OCH_3_**	3.89 (3H, *s*)	3.53 (3H, *s*)	3.56 (3H, *s*)	3.37 (3H, *s*)	2.26 (3H, *s*)	3.80 (3H, *s*)	3.86 (3H, *s*)	3.79 (3H, *s*)
**COOCH_3_**	3.89 (3H, *s*)	3.75 (3H, *s*)	3.88 (3H, *s*)	3.56 (3H, *s*)	3.57 (3H, *s*)	3.86 (3H, *s*)	3.89 (3H, *s*)	3.89 (3H, *s*)
**C-19-OH**								
**N-Me**		3.04 (3H, *s*)						
**N-CHO**							9.30 (1H, *s*)	
**CH=CHPh**	7.85 (1H, *d*)							7.70 (1H, *d*, 16)
**CH=CHPh**	6.88 (1H, *d*)							6.74 (1H, *d*, 16)
			2.4–1.2 (15H, *m*)		2.5–1.2 (14H, *m*)			

* Data not provided; ** The data of the chemical shifts amongst the CH-21 and the aromatic hydrogens were originally exchanged, and were corrected.

**Table 3:** ^13^C-NMR data (in CDCl_3_) for the plumeran indole alkaloids from *Aspidosperma* species.

Carbons				Compound/ *δ*_C_ (ppm)										
	1	5	7	8	9	16	18	20	23	27	36	38	41	43
**2**	167.8	193.0	69.6	64.0	66.0	66.0	69.4	70.3	69.6	71.6	65.4	68.7	63.6	67.6
**3**	51.7	51.8	53.5	53.5	52.8	53.7	53.6	53.7	52.9	53.7	53.6	53.2	52.8	53.0
**5**	50.7	54.4	52.6	52.4	52.8	53.0	52.5	52.4	52.8	52.9	52.7	52.0	52.1	52.2
**6**	44.3	34.9	38.2	38.0	37.1	38.4	37.9	39.4	38.4	38.9	37.9	37.4	39.4	39.3
**7**	55.0	58.1	52.6	52.4	53.7	54.1	52.5	52.2	52.6	52.4	54.3	53.2	53.4	53.5
**8**	138.0	147.2	143.4	128.0	143.3	136.6	125.9	133.1	143.4	137.0	138.9	142.0	140.3	137.4
**9**	121.0	121.7	117.7	115.4	115.3	115.3	115.4	112.4	117.7	122.0	115.4	115.2	115.9	124.3
**10**	120.5	125.3	108.8	125.9	126.2	146.0	125.9	110.0	108.8	117.0	197.8	126.3	124.8	122.3
**11**	127.4	127.6	152.7	110.0	111.8	108.8	111.2	149.4	152.7	127.2	109.6	115.5	111.0	127.9
**12**	109.3	120.2	152.7	148.0	149.5	119.5	*	137.5	152.7	106.4	146.2	149.0	148.6	118.6
**13**	143.4	154.5		141.0	130.1	138.3	*	127.5	129.5	150.5	135.5	129.4	127.8	141.1
**14**	22.2	21.7	21.3	21.5	24.0	21.8	21.6	21.5	24.4	21.9	21.8	21.3	21.6	21.6
**15**	32.9	32.9	34.6	34.1	75.5	34.5	34.2	34.0	75.5	34.4	35.3	42.3	35.1	34.8
**16**	92.8	27.0	24.6	24.7	24.5	28.2	24.4	25.1	25.0	21.6	28.4	24.8	24.8	29.7
**17**	25.6	23.4	22.8	23.0	22.5	23.2	23.1	22.9	24.4	28.8	24.3	34.6	24.3	24.6
**18**	7.3	6.9	5.6	6.7	6.57	6.8	6.6	6.8	6.90	6.7	172.3	175.7	172.0	171.8
**19**	29.3	29.5	29.8	29.9	29.9	29.9	30.1	30.0	30.0	30.0	42.5	45.0	42.4	42.4
**20**	38.2	36.2	35.3	35.4	35.6	35.6	35.5	35.5	35.7	35.5	36.2	35.8	36.1	36.0
**21**	72.7	69.0	71.1	71.0	71.6	71.3	71.1	70.6	71.5	71.1	70.1	69.5	69.4	69.9
**N-COCH_3_**				160.0	171.2		161.4	169.3	171.2			172.0		168.3
**N-COCH_3_**				22.9	23.0			22.7	23.1			23.3		23.2
**11-OMe**			56.0					56.4	56.2					
**12-OMe**			56.0	55.3	53.5	55.2	55.6		56.2		55.4	55.4	55.6	
**15-OMe**					56.3				56.4					
**COCH_2_CH_3_**							28.1							
**COCH_2_CH_3_**							10.1							
**N-CH_3_**										31.4				
**N-CHO**													161.4	
**COOCH_3_**	50.9										50.9	51.1	51.0	51.0
**C** **OOCH_3_**	169.2													

* Data not provided.

## 2. Discussion

The structure of the alkaloid aspidospermine (**8**) has the basic ring skeleton typical of a large number of natural alkaloids. In addition to the characteristic signals for the aromatic methoxyl function, the ^1^H-NMR spectrum shows a very common pattern amongst the indole alkaloidsthe *N*-acetyl group, the C-20 ethyl side chain, and the lone hydrogen atom attached to CH-21, which is not split by any neighboring hydrogen [56].

Thus, the ^1^H and ^13^C-NMR spectra of the alkaloids with the plumeran skeleton present key characteristic signals of the typical aspidospermine pattern. The hydrogen linked at CH-2 shows a signal with a double-doublet multiplicity (due the low sensibility of the 1960’s NMR spectrometers, it was originally characterized as a quartet). This feature indicates that CH-2 hydrogen couples its spin with the two hydrogen atoms at C-16 [56]. The values for chemical shifts of the H-2 vary towards *δ*_H_ 4.5 in the ^1^H-NMR and the CH-2 appears as *δ*_C_ 66 in the ^13^C-NMR spectra.

For the main consulting reference molecule aspidospermine (**8**), the data relative to the hydrogen attached to CH-2 was missing [34] and did not appear on the spectra. Its presence was deduced by the signal of a methine carbon at *δ*_C_ 64 in the ^13^C spectrum. This information was then supplied by another source [35], to the best of our knowledge.

Note that for the alkaloids that present a substituent in C-16, there is a striking difference. Vincadifformine (**1**) is presented here as a precursor of the plumeran skeleton, by the loss of the carbon linked to C16, by a hydrolysis/decarboxylation reaction [21]. For this molecule, the presence of a double bond between C-2 and C-16 justify the absence of the signal of H-2 in the ^1^H-NMR. In the ^13^C-NMR, the chemical shift for C-2 appears at *δ*_C_ 167.8, what may be explained by the presence of the nitrogenous atom and by its conjugation with the carbonyl group, which is also the reason for the chemical shifts of the of the carbomethoxy at *δ*_C_ 169 and the carbon atom C-16 at *δ*_C_ 92.8, which is very characteristic of vincadifformine [30].

Spegazzinine (**3**) and spegazzinidine (**4**) are also substituted in C-16. The signal of H-2 in the ^1^H-NMR spectra appears as a doublet, due to the coupling of the hydrogen attached to CH-2 with the only hydrogen atom in CH-16. The assignments in the ^1^H-NMR spectrum for spegazzinine showed signals for three aromatic hydrogen atoms, an *N*-acetyl function and a C-ethyl group. The consulted bibliography did not provide detailed information regarding the orientation of the aromatic hydrogen atoms [31]. However, the nature of these hydrogens is better known today due to the modern techniques available, and it is now known that there are two doublets attributed to H-9 and H-11, thus revealing *ortho* interactions and one triplet corresponding to H-10. The ^1^H-NMR spectrum of spegazzinidine demonstrated a significant difference only in the region corresponding to aromatic hydrogens; the two aromatic hydrogens have an *ortho* interaction.

The relative configuration of the additional asymmetric center, C-16, in spegazzinine and spegazzinidine was defined because of the constant coupling (*J* = 8.0 Hz), a value typical of axial-axial hydrogens at CH-2 and CH-16 [31].

For 1,2-dehydroaspidospermidine (**5**), the presence of a non-substituted carbon at *δ*_C_ 193.0, assigned as C-2, linked to the indolic nitrogenous atom by a double bond, characterizes it as an indolenine system. As is the case of vincadifformine (**1**), the presence of a double bond between C-2 and N justify the absence of the signal of H-2 in the ^1^H-NMR. The aromatic hydrogens have the expected couplings for a non-substituted indolenine system: a doublet attributed to H-9 with chemical shift of *δ*_H_ 7.53, two multiplets attributed to H-10 and H-11 (each with a chemical shifts of *δ*_H_ 7.25), and a triplet attributed to C-12 with *δ*_H_ 6.17.

Another important feature is the displacement of the chemical shift of the H-12 at non-substituted aromatic ring, as was evident for the demethoxyaspidospermidine (**14**) and 12-demethoxy-*N*-acetylcylindrocarine (**43**). The displacement occurs due to the attenuation of the mesomeric effect of the nitrogenous atom by the presence of an acyl group. This, in addition to the anisotropic effect of the carbonyl group at CH-12 results in the deshielding of the hydrogens at CH-10 and CH-12.

The compounds **9** and **23**, isolated and characterized by Oliveira [32] as new alkaloids, were not reported in the guideline literature [2]. For these two alkaloids, the presence of the methoxy group at CH-15 is deduced by the presence of chemical shifts at *δ*_H_ 3.22 (MeO-15) and at *δ*_C_ 75.5. This is a new substitution pattern observed among the *Aspidosperma* alkaloids.

Hitherto, all the structures have the same ethyl feature at the C-20 side chain. Due to the optical rotation [α]_D_, one- (^1^H-NMR, ^13^C-NMR-{^1^H}and ^13^C-NMR-DEPT or APT) and two-dimensional (homonuclear ^1^H-^1^H-COSY, and ^1^H-^1^H-NOESY; heteronuclear HMQC or HSQC-^1^*J*_HC_ and HMBC-^n^*J*_HC_, n = 2 and 3) NMR experiments, the stereochemistry of this group and the absolute configuration of each of these molecules was well established.

Nevertheless, limaspermine (**28**) and related alkaloids **29** to **35** reveal the oxidative biotransformation of the methyl group present in the carbon atom C-20-ethyl substituent (C-20-CH_2_CH_3_) to yield C-20-CH_2_CH_2_OH, presenting an expressive difference at the lateral chain at C-20 [23,53]. The presence of these moieties in such structures may be recognized by a comparative analysis of the ^1^H-NMR spectra: –CH_2_CH_3_ deduced by triplet signal at *δ*_H_ 0.6 is attributed to the hydrogens of the methyl group (3H-18) and –CH_2_CH_2_OH is characterized by a triplet signal at *δ*_H_ 3.5 corresponding to the carbinolic hydrogens of the methylene CH_2_OH (2H-18). The ^13^C-NMR data for these compounds are not available, so the expected differences of the C-18 and the C-19 in the ^13^C-NMR spectrum can only be predicted, by considering the displacement that occurs to deshielded fields. 

In the alkaloids **36** to **46** (Figure 5) the carbon atom C-20 sustains a –CH_2_COOMe containing a carbomethoxy group (COOMe), which may be explained by the additional oxidative biotransformation of CH_2_CH_2_OH to CH_2_COOH, which is then followed by a methylation reaction. The presence of the C-20-CH_2_COOMe can be recognized by ^1^H and ^13^C-NMR spectra as the anticipated modifications of the ^1^H and ^13^C chemical shifts. The singlet signal corresponding to the hydrogen of the methine carbon CH-21 demonstrates the influence induced by the proximity of the carbonyl group at C-20. This contributes to the signal displacement of the CH-21 hydrogen to *δ*_H_ 2.4 (s), which is different from the observed value of *δ*_H_ 2.23, found for aspidospermine [55]. The most impressive result involves the modifications observed by carbon atoms C-18 and CH_2_-19 in the ^13^C-NMR spectra: *δ*_C_ 171.0 and *δ*_C_ 42.0, respectively.

Data analysis suggests that for the alkaloids **35**, **36** and **38**, the values attributed to aromatics and the CH-21 hydrogens were exchanged. The chemical shifts for the aromatics remain towards *δ*_H_ 6.5–7.0 and for the hydrogen attached at CH-21 remains towards *δ*_H_ 2.2, in accordance with all the other compounds for the same series (**36** to **46**).

In regards to the aromatic hydrogens, it is important to notice that for the structures **42**, **44**, and **46**, the aromatic signals are concerning 8 hydrogens—3 being part of the monosubstituted A ring and the other 5 of a benzyl substituent.

The expected differences associated with the *N*-acyl substituent also appear in the cylindrocarpidine (**39**) and its cynnamoyl derivative *N*-cynnamoyl-19-hydroxycylindrocarine (**46**). They exhibit the absence of the acetyl group and the appearance of lines relative to cynnamoyl system [55]. The coupling constant value (*J* = 16 Hz) observed in the olefinic signals of the ^1^H-NMR spectra was used to confirm the *trans* configuration of the double bond present in the cynnamoyl moiety [50].

## 3. Conclusions

The initial purpose of this work was to emphasize a review of the ^1^H and ^13^C-NMR spectral data for a small group of alkaloids in a discussion that would highlight the spectral differences amongst them. However, we observed that most of the literature data were reported in the 1960’s and the NMR data were incomplete or even unavailable. At that time, the structures of some alkaloids were elucidated on the basis on IR (Infrared) and/or UV (Ultraviolet) and/or MS (Mass Spectrometry) techniques, involving also chemical transformations, before the development of the NMR. Even in the beginning, the NMR spectra presented low sensitivity due to the limited equipment accuracy (frequently about 60 MHz).

Through the modern comparative analysis of ^13^C-NMR-{^1^H}- and DEPT- or APT-^13^C-NMR spectra, it becomes easier to differentiate, classify, and count the signals corresponding to quaternary, methane, methylene and methyl carbon atoms. The DEPTQ (Q = quaternary), appears as a new variation of DEPT for the inclusion of signals corresponding to quaternary carbon atoms. The basic skeleton of the plumeran alkaloids (non-substituted molecules) reveals the presence of four non-hydrogenated (two sp^2^ and two sp^3^), six methine (four sp^2^ aromatics and two sp^3^), eight methylene (all sp^3^) and one methyl carbon atoms, which may be recognized through this comparative analysis. Obviously, the modification of the numbers of CH, CH_2_ and CH_3_ by presence and type of substituent may be characterized with relative facility. This analysis in collaboration with high resolution mass spectrometry significantly contributes to the deduction of a molecular formula. And so, it is possible to classify an unknown alkaloidic molecule in terms of carbon patterns, which would to facilitate the elucidation of its molecular form. Also, the 2D NMR experiments (^1^H-^1^H-COSY, ^1^H-^1^H-NOESY, HMQC or HSQC and HMBC) are especially helpful to solve structural problems, allowing especially assignments of methylene groups that frequently appear in region revealing superimpose [34].

So, this work proposes that not only the chemical shifts for the CH-2 hydrogen (absent in the presence of a double bond between this carbon and the *N*-1*δ*_C_ 193.0 for C-2 as reveled by alkaloid **5**), but also the assignments for the CH-21 and the ethyl group (and its biosynthetic derivatives) sustained by C-20, are the main spectrometric features that characterize the presence of the plumeran alkaloidic skeleton.
